# Usefulness of double plate fixation after failed ORIF for clavicle shaft fracture

**DOI:** 10.1007/s00590-024-03927-5

**Published:** 2024-04-10

**Authors:** Seung Hun Woo, Jung Yun Bae, Sung Won Jung, Min-Hyeok Choi, Suk-Woong Kang

**Affiliations:** 1 Department of Orthopedics, Research Institute for Convergence of Biomedical Science and Technology, Pusan National University Yangsan Hospital, Pusan National University School of Medicine, 20 Geumo-ro, Mulgeum-eup, Yangsan, 626-770 Republic of Korea; 2 Department of Preventive and Occupational & Environmental Medicine, Pusan National University School of Medicine, Pusan National University Yangsan Hospital, Yangsan, Republic of Korea; 3 Office of Public Healthcare Service, Pusan National University Yangsan Hospital, Yangsan, Republic of Korea

**Keywords:** Clavicle, Clavicle shaft, Clavicle mid-shaft fracture, Plate failure, Nonunion, Locking compression plate, Dual plate fixation

## Abstract

**Purpose:**

We aimed to evaluate the clinical and radiological outcomes of double plate fixation for failed clavicle shaft fracture surgery.

**Materials and methods:**

We analyzed 14 patients who underwent double plate fixation due to plate failure after clavicle shaft fracture surgery from March 2016 to March 2021. The study used 3.5 mm locking compression plates for superior clavicle and anterior reconstruction in all patients. In addition, moldable allograft bone was used to fill the bone defect. Clinical and radiological evaluation was performed immediately, at 2 and 4 weeks, and 3, 6, 9, and 12 months postoperatively. The visual analog scale (VAS), University of California at Los Angeles (UCLA) shoulder scale, and American Shoulder and Elbow Surgeons (ASES) scores and range of motion of the shoulder were evaluated as clinical results. For radiological evaluation, anteroposterior, caudal, and cephalad views of both clavicles were used. Successful bone union was defined as complete adjoining of the fracture site through callus formation.

**Results:**

Successful bone union was achieved in all patients, and the mean time to bone union was 16.7 ± 1.2 weeks (range, 12–24 weeks).

Statistically significant improvement in forward flexion and external and internal rotation was observed from 135.5° ± 6.3, 45.2° ± 5.3, and 13° ± 2.3 preoperatively to 157.0° ± 9.3, 68.7° ± 6.3, and 9.8° ± 3.1 at the final follow-up, respectively.

The VAS score improved from an average of 6.2 ± 2.8 preoperatively to 1.3 ± 0.7 at the final follow-up, which was statistically significant (*P* = 0.018). In addition, the ASES score significantly increased from a mean of 52.1 ± 6.3 points preoperatively to 83.6 ± 7.8 points at the final follow-up (*P* = 0.001).

The average UCLA shoulder score was 16.7 ± 1.4 and 31.4 ± 2.2 points preoperatively and at the final follow-up, respectively, which was statistically significant (*P* = 0.001).

**Conclusion:**

Double plate fixation has shown good results after failed open reduction and internal fixation (ORIF) for clavicle shaft fractures. Therefore, in complicated situations after ORIF, double plate fixation is considered a surgical treatment option.

## Introduction

Fractures of the clavicle account for 2.6−10% of all fractures in adults. Ponkilainen Ville et al. derived a pooled incidence rate of 50.3 per 100,000 person-years, investigating eight studies [1]. Clavicle fractures show a bimodal age distribution, i.e., young males < 30 years of age usually due to high energy trauma and older patients aged > 70 years highly due to osteoporosis regardless of sex [2, 3].

In general, conservative treatment shows good results for nondisplaced clavicle shaft fractures, but open reduction and internal fixation (ORIF) are commonly applied for patients with displaced or comminuted mid-shaft clavicle fractures [4, 5]. Although open reduction and plate fixation have the advantages of a lower nonunion rate (0−3%) and earlier recovery than conservative treatment (21%), a risk of construct failure such as plate breakage, bending of the plate, or screw loosening is associated with this surgical method [6–8]. According to recent studies, construct failure rates have been reported from 1.2 to 12.6% [9–11].

Construct failure and nonunion are caused by complex biological factors related to age and osteoporosis and mechanical factors related to inappropriate fixation [12]. Therefore, if clavicle nonunion and plate breakage occur, a better bone healing environment should be created by using more rigid fixation. According to a biomechanical study, the addition of an anterior mini-plate to traditional superior plating improved construct stiffness compared to that of single superior plating [6].

Therefore, in this study, we retrospectively evaluated the effectiveness of double plate fixation after failed ORIF for clavicle shaft fracture.

## Materials and methods

The Institutional Review Board exempted the study from review given the retrospective design of the study. From March 2016 to March 2021, a total of 16 patients underwent double plate fixation due to plate failure after clavicle shaft fracture surgery. Two patients were not followed up for > 2 years; therefore, a total of 14 patients were analyzed.

The mean age of the 14 patients (9 males, 5 females) was 53.42 years (22−78 years), and the mean follow-up period was 26.4 months (24−36 months). Four patients underwent primary plate fixation at our hospital, with early plate breakage occurring in two patients, and refracture after metal removal experienced in two patients. Ten patients were transferred from other hospitals after primary surgery, including three patients with early plate breakage, six with plate breakage with nonunion, and one patient with screw loosening with nonunion (Table 1).

**Table 1:** Patients demographics

Case	Age (years)	Sex	Side	Type of fractures (Robinson)	Etiology	Revision (months)
1	M	62	R	Type 2B1 (wedge)	Early plate breakage	5
2	M	57	L	Type 2B1 (simple)	Early plate breakage	3
3	M	22	R	Type 2B1 (wedge)	Plate breakage with nonunion (atrophic)	24
4	M	78	L	Type 2B2 (comminuted)	Early plate breakage	6
5	F	77	R	Type 2B1 (wedge)	Plate breakage with nonunion (atrophic)	18
6	F	63	R	Type 2B2 (comminuted)	Screw loosening with nonunion (hypertrophic)	10
7	M	48	L	Type 2B1 (wedge)	Refracture after metal remove	2
8	F	49	L	Type 2B1 (wedge)	Refracture after metal remove	1
9	M	32	L	Type 2B1 (simple)	Plate breakage with nonunion (hypertrophic)	12
10	M	68	R	Type 2B2 (comminuted)	Early plate breakage	3
11	F	57	R	Type 2B1 (wedge)	Plate breakage with nonunion (atrophic)	13
12	M	53	R	Type 2B1 (wedge)	Plate breakage with nonunion (atrophic)	10
13	M	34	L	Type 2B1 (simple)	Early plate breakage	4
14	F	48	L	Type 2B1 (wedge)	Plate breakage with nonunion (hypertrophic)	11

**Fig. 1:**
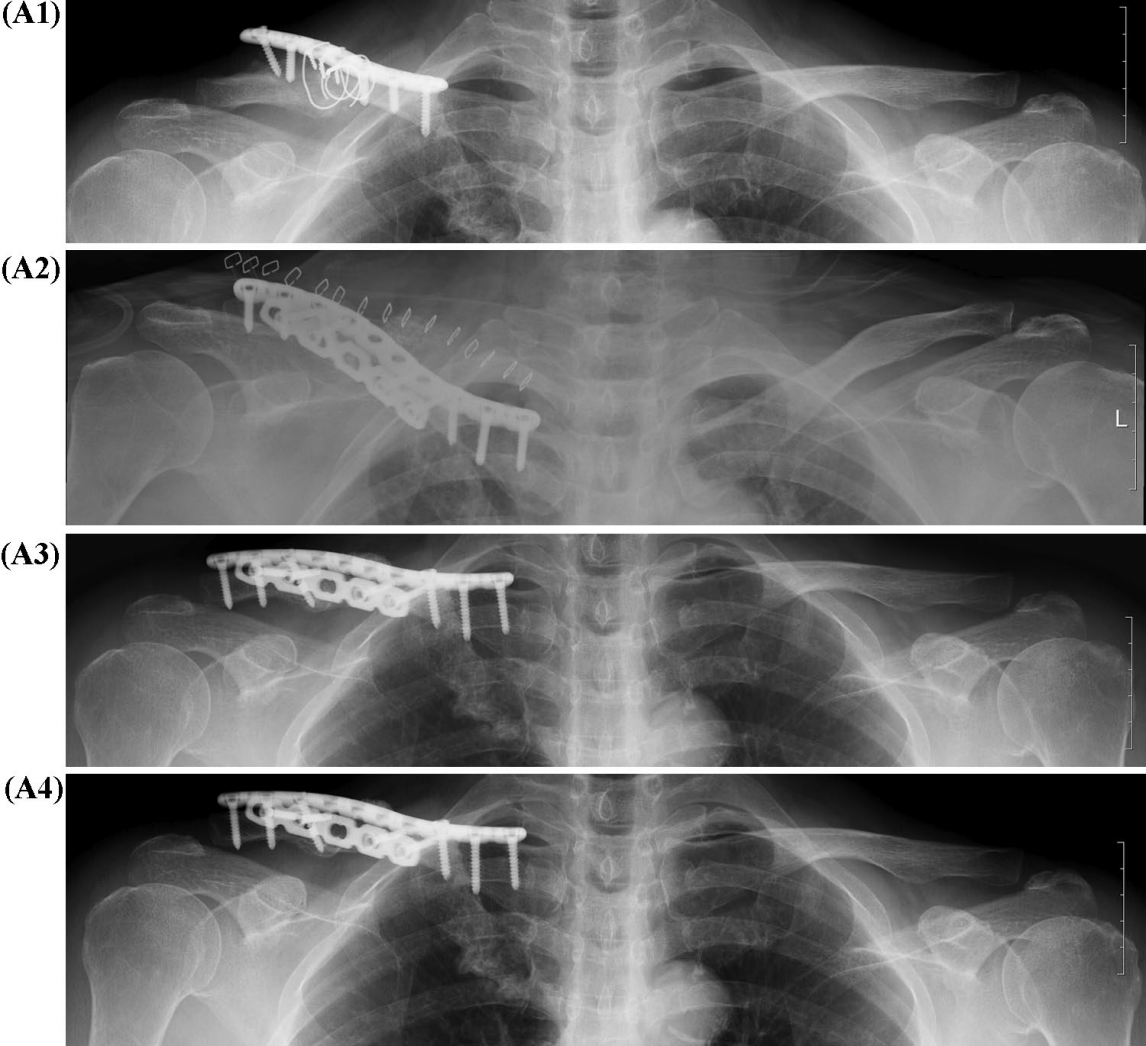
A 63-year-old female who underwent ORIF for clavicle shaft fracture 10 months earlier was transferred due to plate and screw loosening with nonunion. **A1** Plate and screw loosening after primary ORIF, **A2** postoperative anteroposterior view of double plate fixation, **A3** anteroposterior view 3 months after revision surgery, **A4** anteroposterior view 24 months after revision surgery

All surgeries were performed by one surgeon. Under general anesthesia, the patient was placed in the beach chair position. The fracture site was exposed via the previous incision. The fibrotic tissue in the fracture area was removed, and in the case of nonunion, the sclerotic bone and cyst were removed using a burr or curette. We used 3.5 mm superior locking compression plates (LCPs) for superior clavicle and anterior reconstruction (Depuy Synthes, MA, USA) in all patients. In addition, bone allograft was not performed, and the defect was filled using moldable allograft bone (S1-OXB, MedPark). Alignment was adjusted using reduction forceps, and the superior locking plate was fixed first. Using a 9−10-hole-long plate fixation, three locking and cortical screws were inserted at each end, and then, the bone graft was performed on the defect. Then, to fix the anterior aspect of the clavicle, the reconstruction plate was bent according to the bone curvature, and a screw was inserted (Fig. 1).

Clinical and radiological evaluation was performed immediately, at 2 and 4 weeks, and 3, 4, 6, 9, and 12 months postoperatively. The visual analog scale (VAS), University of California at Los Angeles (UCLA) shoulder scale, and American Shoulder and Elbow Surgeons (ASES) scores were evaluated as clinical results. The range of motion was defined as forward flexion in the scapular plane and external rotation with the elbow at the side. Internal rotation was determined by measuring the highest spinal segment the patient could reach with the thumb pointed upward. To facilitate statistical analyses, the spinal segment level was converted to continuous numbers: T1–T12 levels were represented by 1 through 12; L1–L5 levels were represented by 13 through 17; and the sacrum was represented by 18 [13]. For radiological evaluation, anteroposterior, caudal, and cephalad views of both clavicles were performed regularly during follow-up. Bone union was defined clinically as lack of tenderness and radiological evidence of callus at the fracture site. The ratios of the length of the fracture site and angulation to the length and angulation of the opposite side were measured using anteroposterior views of both clavicles [14, 15].

All statistical analyses were performed using SPSS Statistics for Windows, version 17.0 (SPSS Inc., Chicago, IL, USA). The nonparametric Wilcoxon signed-rank test was used to analyze the data. The significance threshold was set at 0.05.

## Results

Successful bone union in all patients was achieved, and the mean time to bone union was 16.7 ± 1.2 weeks (range, 12–24 weeks).

The preoperative mean forward flexion and external and internal rotation were 135.5° ± 6.3, 45.2° ± 5.3, and 13° ± 2.3, and at the final follow-up, the averages were 157.0° ± 9.3, 68.7° ± 6.3, and 9.8° ± 3.1, showing statistically significant differences, respectively.

A statistically significant improvement was observed in the VAS score from 6.2 ± 2.8 preoperatively to 1.3 ± 0.7 at the final follow-up (*P* = 0.018). In addition, the ASES score significantly increased from a mean of 52.1 ± 6.3 points preoperatively to 83.6 ± 7.8 points at the final follow-up (*P* = 0.001).

The average UCLA shoulder score was 16.7 ± 1.4 points preoperatively and 31.4 ± 2.2 points at the final follow-up, showing a statistically significant difference (*P* = 0.001) (Table 2).

**Table 2:** Clinical outcomes before and after surgery

Variable	Preoperative	Last follow-up	*P*-value
Range of motion			
Forward flexion	135.5° ± 6.3	157.0° ± 9.3	0.023
External rotation	45.2° ± 5.3	68.7° ± 6.3	0.002
Internal rotation^1^	13 ± 2.3	9.8 ± 3.1	0.001
VAS score	6.2 ± 2.8	1.3 ± 0.7	0.018
ASES score	52.1 ± 6.3	83.6 ± 7.8	0.001
UCLA score	16.7 ± 1.4	31.4 ± 2.2	0.001

Qualitative variables were expressed as the mean ± standard deviation

^1^Measured by the vertebral level that the patient was able to reach with the thumb and numbered serially as 1 to 12 for the 1st to 12th thoracic vertebrae, 13 to 17 for the 1st to 5th lumbar vertebrae, and 18 for any level below the sacral vertebrae

The average ratio of the length of the fractured clavicle to the contralateral clavicle was 0.83 ± 0.04 before surgery and 0.97 postoperatively, which was statistically significant (*P* = 0.000). Additionally, the angulation of the fractured area was significantly improved from 2.50 ± 1.16 before surgery to 1.10 ± 0.12 postoperatively (*P* = 0.002) (Table 3).

**Table 3:** Radiological outcomes after surgery

Variable	Preoperative	Last follow-up	*P*-value
Ratio to the length of the opposite side	0.83 ± 0.04	0.97 ± 0.02	0.000
Ratio to the angulation of the opposite side	2.50 ± 1.16	1.10 ± 0.12	0.002

## Discussion

We performed superior and anterior double plating in patients with failed open reduction for clavicle mid-shaft fracture, and successful bone union was obtained in all patients. Additionally, pain and functional scores showed improvement postoperatively.

Mid-shaft clavicle fractures have generally shown good results with both conservative and surgical treatments [4, 5]. Conservative treatment is preferred for nondisplaced fractures; however, surgical treatment is preferred for fractures that have been displaced or shortened by more than 2 cm [16]. Various surgical treatments such as intramedullary nail fixation, pin fixation, plate fixation using the minimally invasive plate osteosynthesis technique, and plate and screw fixation (superior plating or anteroinferior plating) have been introduced for displaced clavicle fractures, but a superior LCP is the most commonly used surgical method [3, 16–18]. The Superior LCP® (Depuy Synthes, MA, USA) shows good results, but various complications such as nonunion, plate deforming, plate breakage, screw loosing, infection, and brachial plexus injury have been reported. Especially, plate failure due to osteoporosis or inappropriate fixation has been reported in approximately 5−7% of cases [9, 11]. A total of 61 patients in our hospital underwent primary open reduction and plate fixation for displaced mid-clavicular shaft fractures during the study period. Successful bone union occurred in all patients; however, early plate breakage occurred in 2 patients (3.2%) who were males aged 57 and 68, respectively, without osteoporosis. Both patients had no pain from the 2nd week postoperatively and no evidence of antecedent trauma; however, the plate breakage apparently occurred during excessive exercise.

The occurrence of refracture after plate removal after bone union is a rare event. Zhu, Yurun et al. reported a 6.5% incidence rate of refracture, and risk factors included Robinson type 2B2 clavicular fracture, fair/poor reduction, and postmenopausal females with a short interval (< 12 months) from primary surgery to implant removal [18, 19]. Plate removal was performed in a total of 40 patients in our hospital, and refracture occurred in two patients (5%). No specific issues were encountered during plate removal; however, two patients had progressive postoperative pain, and refracture occurred during activities of daily living. These two patients had no unusual finding on the preoperative radiograph, and the plate was removed at 1 year after primary surgery.

When considering a second surgery, the use of more rigid fixation and bone graft is necessary. According to a recent biomechanical study, the addition of an anterior mini-plate to a traditional superior plate demonstrated higher construct stiffness and strength compared to those using a single superior or double orthogonal plate [6]. Therefore, we have been conducting double plating since 2018 with good clinical and radiological results.

There are several limitations of this study. First, because of the retrospective design and only a small cohort of 14 patients was involved, the validity of the study may be questionable. The low number of patients in our study was attributed to the fact that no cases of nonunion were treated conservatively at our hospital, and the frequency of complications after open reduction and plate fixation was small. However, our study was performed by a single surgeon in a single center using the same surgical technique. Although it was a retrospective study, postoperative evaluations were conducted as planned, and 14 patients were followed up for > 2 years. Second, diagnoses among the cohort varied. However, our study examined the utility of double plate fixation after failed open reduction and plate fixation for clavicle shaft fractures. Third, despite using computed tomography for confirmation, callus formation was not clearly observed due to the interference from the presence of the double plate. However, one side of cortical continuity could be confirmed, viewing it from various angles. Furthermore, clinically, patients had no pain at the fracture site and did not report any restrictions of activity. Thus, the definition of bone union was satisfied [17]. One patient underwent implant removal surgery, and refracture did not occur until the following year. Additionally, we used moldable allografts. However, evaluating the effectiveness of bone grafts is difficult because quality after bone union cannot be confirmed for the same reasons given above.

## Conclusion

Double plate fixation has shown good results after failed open reduction and plate fixation for clavicle shaft fractures. Therefore, in complicated situations after ORIF, double plate fixation is considered a good option.
