# Correction to: Usefulness of double plate fixation after failed ORIF for clavicle shaft fracture

**DOI:** 10.1007/s00590-024-04024-3

**Published:** 2024-07-12

**Authors:** Seung Hun Woo, Jung Yun Bae, Sung Won Jung, Min-Hyeok Choi, Suk-Woong Kang

**Affiliations:** 1 Department of Orthopedics, Research Institute for Convergence of Biomedical Science and Technology, Pusan National University Yangsan Hospital, Pusan National University School of Medicine, 20 Geumo-ro, Mulgeum-eup, Yangsan, 626-770 Republic of Korea; 2 Department of Preventive and Occupational & Environmental Medicine, Pusan National University School of Medicine, Pusan National University Yangsan Hospital, Yangsan, Republic of Korea; 3 Office of Public Healthcare Service, Pusan National University Yangsan Hospital, Yangsan, Republic of Korea

**Correction to: European Journal of Orthopaedic Surgery & Traumatology** 10.1007/s00590-024-03927-5

In the original version of this article, in the “Materials and methods” section, sixth sentence of the third paragraph which previously read

In addition, bone allograft was not performed, and the defect was filled using moldable allograft bone (S1-OXB, MedPark).

Should have read

Additionally, bone autograft was not performed and the defect was filled using moldable allograft bone (S1-OXB, MedPark).

